# Different Views about the Nature of Gender-Related Asymmetries in Tasks Based on Biological or Artefact Categories

**DOI:** 10.3233/ben-2009-0247

**Published:** 2010-06-29

**Authors:** Guido Gainotti, Francesca Ciaraffa, Maria Caterina Silveri, Camillo Marra

**Affiliations:** ^1^Center for Neuropsychological Research of the Policlinico Gemelli/ Catholic University of RomeRomeItaly; ^2^CEMIPoliclinico Gemelli/ Catholic University of RomeRomeItaly

**Keywords:** Sex differences, living beings, familiarity, social roles, perceptual processing, sources of knowledge

## Abstract

Sex-related asymmetries in the ability to process different semantic categories have been reported both in normal subjects and in brain-damaged patients, but the nature of these asymmetries is still controversial. Some authors suggest that these differences might be due to social-role related familiarity factors, whereas others attribute them to inborn neural differences rooted in evolution. Drawing in part on this second line of thought, some authors have suggested that gender-related asymmetries might be due to differences in stimulus processing between men and women, namely, to the tendency of females to focus mainly on perceptual features and of males to focus equally on both perceptual and functional features. To test this hypothesis, we asked 53 male and 65 female undergraduate students to evaluate the relevance of a number of perceptual and functional features in the representation of various kinds of biological and artefact categories. Contrary to the hypothesis, evaluation of the weight of different sources of knowledge in representing living and artefact categories was similar in males and females.

## 1. Introduction

A sizable body of data, recently gathered both in normal subjects and in brain-damaged patients, suggests there are sex-related asymmetries in the ability to process different semantic categories.

For example, significant and consistent asymmetries have been observed in normal subjects on speeded naming [22] and identification [23] tasks, on name generation tasks [25], on semantic fluency tasks [8,9], and on object decision tasks [3]. Similar asymmetries have also been found in normative naming data from adults and children [15,29], by evaluating familiarity ratings for various categories [1] and by studying the age of acquisition of common names [2,15]. Furthermore, using the semantic priming paradigm, Bermeitinger et al. [4] recently showed that sex differences exist for biological vs artefact categories. Analogous asymmetries have also been found in brain-damaged patients [10,16,19,21, 26,28,31], in whom a relative sparing of the categories showing a sex-related advantage in normal subjects has usually been observed.

Nevertheless, the exact pattern of the categories more easily processed by men and women and the nature of these gender-related asymmetries are still controversial. Indeed, most authors (e.g. [3,9,19,21–27]) report that males fare better with artefacts and that females process biological stimuli more proficiently. Other authors, however, have argued that the pattern of gender-related asymmetries is more complex than the simple distinction between biological and artefact categories, because men show an advantage for some living categories (such as ‘animals’) and women for some artefact categories (such as ‘furniture’ and ‘kitchen utensils’).

For instance, several authors found a sex-related dissociation in the biological categories between ‘animals’

ISSN 0953-4180/09,10/$27.50 © 2009/2010 – IOS Press and the authors. All rights reserved and ‘plant life’. Indeed, in McKenna and Parry’s [29] normative study women were better at naming fruits and vegetables, whereas men were better at naming animals. Similar results were recently obtained by Barbarotto et al. [2], who, studying the naming latencies of normal adults, found that women were significantly faster in naming fruit and vegetables, whereas men were marginally faster in naming animals. Furthermore, normal women fared better with fruits, but not with animals, on semantic fluency tasks [8,9,25] and naming tasks [24] and gave higher familiarity ratings to fruits and vegetables but not to animals [1]. A similar sex-related dissociation between ‘animal’ and ‘plant life’ categories was observed in brain-damaged patients by Gainotti [16] and by Moreno-Martinez et al. [31]. In a review of patients with a category-specific semantic disorder, the former found that men were systematically more impaired with fruits and vegetables and women were more impaired with animals. The latter observed that men performed better with animals, but not with fruits and vegetables, on a category fluency task administered to a group of Alzheimer disease patients.

If we pass from the pattern of biological categories more easily processed by men and women to the origins of these gender-related asymmetries, we see that two general models have been offered to account for these differences. The first interpretation is based on the ‘domains of knowledge hypothesis’ [10–13,31], which assumes that natural selection produced specialized and therefore dissociable neural circuits for animals and plant life, because these two biological categories play a different but equally important role in human survival. An interpretation of sex-related asymmetries coherent with this general hypothesis was offered by Laws [23, 25] and refined by Laiacona et al. [20]. According to Laws [23,25], it is conceivable that the main subsistence activities of men (hunting) and women (gathering) produced a greater development of brain circuits dealing with tools and animals in men and with fruits and vegetables in women. In a similar vein, Laiacona et al. [20], proposed that the evolutionary pressures which prompted the development of different brain networks dedicated to animals and plant life might also have provided each gender with more efficient cognitive representations of their main foraging targets (i.e. animals for men and fruits and vegetables for women). A different interpretation of gender-related asymmetries was proposed by Gainotti [16] based on his review of the influence of gender and lesion location on naming disorders for animals, plant life and artefacts. This author suggested that only the distinction between living and non living things reflects an inborn anatomically-based categorical organization, whereas the discrepancy (within living entities) between animals and plant life might be due to social roles related familiarity factors, namely, to men’s greater familiarity with animals and women’s with fruits and vegetables. According to this hypothesis, sex-related asymmetries should not be due to inborn biological factors, but to social-cultural factors such as the different familiarity of males and females with the items in these different categories. One problem with these alternative theoretical models is that they can hardly be submitted to empirical testing. We thought that results of a paper recently published by Bermeitinger et al. [4], using the semantic priming paradigm to study retrieval processes with biological and artefact categories in men and women could perhaps allow to check the inborn differences hypothesis. In this study, females showed positive priming effects for natural categories and negative priming effects for artefact categories, whereas males showed positive priming effects for both categories. As some authors (e.g. [14,36]), have suggested that biological categories might be mainly based on perceptual features and that artefact categories might rely more on functional features, Bermeitinger et al. [4] proposed that sex asymmetries might be due to a difference in stimulus processing between men and women, with a tendency of the latter to focus mainly on perceptual features. To test this hypothesis, we administered stimuli belonging to various domains of living and artefact categories to normal young male and female university students and asked them to evaluate the relevance that, in their knowledge of those items, had played a number of perceptual (visual, auditory, olfactory gustatory and tactual) and motor activities and of language-mediated encyclopaedic information. If Bermeitinger et al.’s [4] hypothesis is correct, it could be logical to expect that females will give greater value to perceptual features they preferentially rely on to analyse conceptual categories. Therefore, the following predictions can be made:

A) Women should give greater value to perceptual (visual, auditory, gustatory and olfactory) features than to functional somato-sensory inputs, motor activities and language-mediated information, whereas men should give a more balanced evaluation of functional and perceptual features. We decided to consider visual, auditory, gustatory and olfactory information as ‘perceptual’ and somato-sensory inputs, motor activities and language-mediated information as ‘functional' for the following reasons: 1) Some authors, e.g. [5–7], drawing on a suggestion originally offered by Warrington and McCarthy [35], distinguished within functional knowledge the function of an object from its manipulation; 2) somato-sensory inputs and motor activities are intrinsically related in manipulatory functions; 3) all language-mediated (namely, functional, encyclopaedic, etc.) features were contrasted with sensory properties in Caramazza and Shelton's [13] study, which aimed at checking the assumption of a differential weighting of sensory and functional information in the representation of knowledge about living things and artefacts; the same logic has been followed by other authors; 4) in a recent study by Vigliocco et al. [34], which aimed at gathering data for conceptual feature representations from various conceptual domains, 'animals', and 'fruits and vegetables' showed a higher average proportion of visual and other perceptual features, whereas artefacts showed a greater average proportion of functional and motor features.

B) The suggested tendency of women to pay greater attention to perceptual information and of men to give a more balanced evaluation of functional and perceptual features should be particularly valid for the biological categories, in which the effects that prompted Bermeitinger et al.'s [4] hypothesis were more evident.

## 2. Experimental procedure

### *2.1. Subjects and materials*

#### *2.1.1. Participants*

The study was conducted on 118 undergraduate students (53 males and 65 females) from the Faculty of Medicine of the Catholic University of Rome.

#### *2.1.2. Material*

Data were collected using a booklet that contained 75 sheets of paper in a standardized format; a different item headed each sheet. Each item was represented in the corresponding sheet with both a picture and a written word printed in big capital letters under the picture. The items consisted of 28 living things (6 domestic animals, 6 wild animals, 6 flowers, 5 vegetables and 5 fruits) and 21 artefacts (5 pieces of furniture, 5 vehicles, 5 articles of clothing and 6 tools). A list of these items has been reported in Appendix 1. The original protocol, which has been described in a twin paper [17], also included 26 unique entities (13 famous people, 6 famous monuments and 7 famous towns) that will not be taken into account in this paper because they are not relevant to the issue being considered here. Items were pseudo-randomized so that stimuli belonging to the same category never were printed on successive sheets. For each stimulus, subjects were requested to evaluate the familiarity that they had with that item and the relevance that, in constructing our knowledge of that object, could have a number of perceptual (visual, auditory, tactual, olfactory and taste perceptions) and motor activities as well as language-mediated functional-encyclopaedic information. In the instructions given to our subjects, the term 'language-mediated encyclopaedic information' was defined as denoting all those features (such as functional use of objects, abstract definitions, metaphorical expressions and so on) that are learned through language, but not through direct or media-mediated sensory-motor information.

To indicate the familiarity that they had with each stimulus and the relevance of each 'source of knowledge', subjects had to assign to each item a series of scores ranging between 0 and 7, where 0 denoted 'no familiarity' and 'no relevance' and 7 'very high familiarity' and 'very high relevance'. An instance of a response sheet given to participants, asking them to indicate their familiarity with each stimulus and to evaluate the relevance that in their mental representations could play a number of 'sources of knowledge' is reported in Appendix 2.

Five practice sheets, containing items drawn from 5 different categories, were given to the students to explain the kind of evaluation they should make. No subject had difficulty understanding the task, evaluating how familiar each stimulus was or indicating the scores corresponding to the relevance of each 'source' of knowledge on the response sheets. However, some subjects tended to give overall low or high scores on all items pertaining to each source of knowledge. For instance, for the item 'tomato' almost all subjects gave the highest score to its visual properties (red colour), its taste and to the actions made to cook it, considering verbal descriptions as less important and auditory sensations as quite irrelevant. However, in some subjects this profile was displaced toward the right (upper scores), whereas in other subjects it was displaced toward the left (lower scores). For example, when evaluating the sources of knowledge of the item 'tomato'

one subject gave 7 points to its visual properties, 6 to taste and cooking actions, 5 to language-mediated functional-encyclopaedic information, 4 to tactual, 3 to olfactory and 1 to auditory perceptions), whereas an other subject gave 4 points to the visual properties, 3 to the taste and the actions made to cook it, 2 to the tactual perceptions and language-mediated information and to olfactory information and 0 to auditory perceptions). It is clear that raw scores were different among subjects but that the weight they assigned to each source of knowledge was quite similar. To equalize these evaluations, we transformed for each item the raw scores assigned to each source of knowledge into percentage scores, using the following formula:

$$\frac{\text{specific source of knowledge}}{\text{sum of all source of knowledge}} \times 100$$

For instance, in the first previously reported case, the sum of all the putative sources of knowledge was 32 (7+6+6+5+4+3+1) and the percentage values of the various sources of knowledge were 22% (7/32) for vision, 19% (6/32) for taste and cooking actions, 16% (5/32) for language-mediated information, 12% (4/32) for tactual, 9% (3/32) for olfactory and 3% (1/32) for auditory perceptions. In the second previously reported case, the total value of the putative sources of knowledge was 16 (4+3+3+2+2+2+0) and the percentage values of the various sources were 25% (4/16) for vision, 19% (3/16) for taste and cooking actions and 12% (2/16) for tactual and olfactory perceptions and for language-mediated information. In this manner, the rank orders as well as the % values of the responses given by the two subjects were very similar, though the absolute values were rather different. This transformation of raw scores into % scores allows us to understand why the scores reported in Table 2 (corresponding to judgments made by males and females about the relevance of the various sources of knowledge for the biological and artefact categories) do not range from 0 to 7 (raw scores given by each subject) but from 0 to 100 (percentage of relevance attributed by each subject to each source of knowledge).

### 2.2. *Statistics*

In the first analysis, concerning familiarity, data were treated by means of a two-way ANOVA, considering gender and general domains of the biological and artefacts categories as independent variables and familiarity as the dependent variable. Post hoc comparisons among the various biological and artefact categories, were computed by means of independent 't' tests, followed by Bonferroni's correction for multiple comparisons.

In the second analysis, concerning the evaluations of relevance of the various 'sources of knowledge', the prevalence of the perceptual and functional sources of knowledge in males and females was analysed separately for the individual categories by means of a one-way MANOVA and post-hoc comparisons among the various sources of knowledge, across the biological and artefact categories, were computed by means of paired 't' tests, followed by Bonferroni's correction for multiple comparisons.

## 3. Results

### 3.1. *Judgments made by males and females of the personal familiarity that they had with the items composing the various subcategories of living things and of artefacts*

Table 1 reports the mean values (and the corresponding SD) of the judgments made by males and females about the personal familiarity that they had with the various subcategories of living things and artefacts, the results of the two-ways ANOVA and the values of the individual paired 't' tests.

Data reported in Table 1 show that no interaction can be found between 'categories' and 'gender', because the results of the two-ways ANOVA showed significant differences for the main factors 'categories' ($F_{(8-80)} =$ 19.189; $p <$ 0.00001) and 'gender' ($F_{(1-80)} =$ 6.344, $p <$ 0.01376), but no interaction between these factors ($F_{(8-80)} =$ 0.220; $p <$ 0.98). As for the main factor 'gender', females tended in general to give higher familiarity judgments, whereas with respect to the 'categories' factor, some differences were observed among the categories ( familiarity being higher for 'furniture', 'clothes' and 'fruits' and lower for 'wild animals' and 'vehicles'). Furthermore, the values of the individual paired 't' tests for each category failed to show any difference, confirming that there was not a prevalence of familiarity for a specific category in males or females.

### 3.2. *Judgments made by males and females of the relevance of the various sources of knowledge for the biological and artefact categories*

Results were assessed by matching first the evaluations made by males and females for each catego-

Table 1
Mean values (and SD) of the judgments made by males and females of the personal familiarity that they had with the various subcategories of living things and artefacts

| | | Females | Males | 't' tests |
|---|---|---|---|---|
| Fruits | | 6.45 (0.15) | 6.25 (0.24) | 0.15 |
| Vegetables | | 6.25 (0.24) | 6.09 (0.28) | 0.23 |
| Flowers | | 5.64 (0.62) | 5.4 (0.66) | 0.61 |
| Pets | | 5.55 (0.42) | 5.46 (0.38) | 0.41 |
| Wild animals | | 5.21 (0.29) | 4.76 (0.27) | 0.42 |
| | *Biological* | *5.82 (0.58)* | *5.59 (0.68)* | *0.17* |
| Furniture | | 6.54 (0.16) | 6.37 (0.22) | 0.15 |
| Clothes | | 6.44 (0.22) | 6.24 (0.22) | 0.21 |
| Vehicles | | 5.89 (0.52) | 5.71 (0.72) | 0.52 |
| Tools | | 6.33 (0.35) | 6.23 (0.40) | 0.34 |
| | *Artefact* | *6.30 (0.40)* | *6.14 (0.47)* | *0.33* |

Table 2
Evaluations of relevance about the various 'sources of knowledge' made by males and females for members of biological and artefact categories

| | Males | | Females | |
|---|---|---|---|---|
| Sources of knowledge | Artefacts | Biological | Artefacts | Biological |
| Visual (*) | 21.29 (1.63) | 20.47 (2.64) | 22.61 (1.70) | 21.28 (2.73) |
| Auditory | 12.09 (2.27) | 11.20 (4.09) | 11.62 (3.27) | 10.57 (4.13) |
| Olfactory (**) | 10.01 (2.61) | 14.37 (3.10) | 9.86 (2.58) | 14.28 (2.86) |
| Gustatory (**) | 6.67 (1.56) | 11.5 (4.86) | 5.75 (1.91) | 11.08 (5.99) |
| Tactual (**) | 15.41 (1.84) | 13.43 (1.80) | 16.88 (2.82) | 13.85 (2.74) |
| Action (**) | 19.61 (2.06) | 13.16 (1.81) | 19.28 (1.86) | 13.78 (1.53) |
| Language | 14.90 (1.13) | 15.85 (4.36) | 14.0 (2.08) | 15.13 (4.20) |

$^{**}P < 01$, $^{*}p < 0.05$ for Artefacts vs Biological categories both in men and women. The standard deviations are reported in parenthesis.

ry, considering separately each perceptual, motor or language-mediated source of knowledge and grouping in a second time evaluations based on perceptual and functional features. Analytical evaluations of relevance attributed to visual, auditory, olfactory and gustatory perceptions and to motor, tactual and language- mediated source of knowledge are reported in Table 2.

The two-way MANOVA revealed a slightly significant effect for the independent factor 'gender' (Wilk's lambda $_{(7,88)}$: 0.81; $p < 0.03$). The analysis of the single F showed that the smooth difference between males and females was mainly due to a specific effect observed for the 'visual' source of knowledge (F: 5.06; $p < 0.026$ – $\eta^2$: 0.05), where females prevailed over males irrespectively of the category (biological or artifact) considered. A highly significant effect was found for the independent factor 'categories' (biological vs artifacts: Wilk's lambda$_{(7,88)}$: 0.18; $p < 0.0001$).

In particular specific differences in the processing of biological and artifact categories were found for 'visual' (F: 5.16; $p < 0.026$ – $\eta^2$: 0.065), 'olfactory' (F: 58.1; $p < 0.0001 - \eta^2$: 0.38) and 'gustatory' (F: 33.65; $p < 0.0001$ $-\eta^2$: 0.26) information (considered as more important for the biological entities) and for tactual (F: 27.28; $p < 0.0001$ $-\eta^2$: 0.21) and motor (F: 262.97; $p < 0.0000$ $-\eta^2$: 0.45) sources of knowledge (which prevailed in artifacts). No differences in the evaluation of biological and artifact categories were observed for auditory and encyclopedic language-mediated sources of knowledge. Finally no significant interaction was found between the two main factors (Wilk's lambda $_{(7,88)}$: 0.82; $p = 0.57$), suggesting that no specific prevalence of a source of knowledge for a specific category could be detected in men and women. Consequently, the relevance that different 'sources of knowledge' have in the representation of biological and artefact categories should be considered quite similar in males and females.

As for the comparisons among the various sources of knowledge across the biological and artefact categories, multiple paired 't' tests with Bonferroni correction were performed by adjusting the significance level accordingly ($p < 0.05 = p < 0.0014$). Analyses showed: (a) that visual information represented the most important of all the sources of knowledge considered; (b) that what distinguishes living from artefact categories is the pattern of sensori-motor information associated with the visual data, rather than the weight of perceptual vs. functional features, because in the case of biological entities visual information was mainly associated with other perceptual features, whereas in the case of artefacts visual data were mainly integrated with action-related information.

### 3.3. *Evaluation of the hypothesis which assumes that women give greater weight to perceptual information and that men make a more balanced evaluation of functional and perceptual features*

The data reported in Table 2, showing that men and women make very similar evaluations of the weight that different 'sources of knowledge' have in the representation of biological and artefact categories, are clearly at variance with the Bermeitinger et al.'s [4] hypothesis. However, to make a stricter test of this hypothesis we grouped as 'perceptual' all evaluations concerning visual, auditory, gustatory and olfactory information and as 'functional' the sum of somato-sensory, motor and language-mediated information and we evaluated whether an interaction could be found between gender and weight attributed to perceptual vs functional information. This analysis was made taking into account separately in Fig. 1 the items belonging to the biological and artefact categories.

The data reported in these figures consistently show that men and women make very similar evaluations of the weight that different 'sources of knowledge' have in the representation of biological (Wilks' lambda$_{(2-53)}$: 0.93; $p = 0.15$) and artefact (Wilks' lambda$_{(2-39)}$: 0.98; $p = 0.92$) categories. Moreover, our whole sample (irrespective of gender) attributed equal relevance to perceptual and functional features in the representation of living beings ('t' test: 0.65; $p = 0.52$) and greater weight to 'functional' information in the representation of artefacts ('t' test: 18.74; $p = 0.0001$).

## 4. Discussion

The main purpose of the present study was to check the Bermeitinger et al.'s [4] hypothesis that gender-related differences in the ability to process different semantic categories may not be simply due to familiarity factors, but rather to a tendency of females to focus mainly on perceptual features and of males to focus equally on perceptual and functional features.

To evaluate this hypothesis we recruited two groups of undergraduate students (one comprised of males and the other of females) and asked them to evaluate the relevance that a number of perceptual (visual, auditory, olfactory and taste perceptions) and functional (tactual, motor and language-mediated) types of information could have in our knowledge of various kinds of biological and artefact categories. The Bermeitinger et al.'s [4] hypothesis was not confirmed, because evaluation of the weight of different 'sources of knowledge' in the representation of biological and artefact categories was very similar in males and females. In fact, participants of both genders maintained the following: (a) that visual data must be considered as the main source of knowledge for both biological and artefact categories, (b) that these data are associated with different sources of knowledge in the case of biological and artefact categories. In the case of living entities visual information converges with other perceptual features, whereas in the case of artefacts it is primarily integrated with action-related information.

Data concerning our subjects' general evaluation of the weight that different sources of knowledge have in the mental representation of biological and artefact categories have already been discussed in our previously mentioned twin paper [17]; therefore, we will not discuss this topic here. What is important with respect to the aims of the present research is that the evaluation of the weight of 'perceptual' and 'functional' features in the representation of biological and artefact categories was very similar in males and females, because in the representation of biological entities both gender groups attributed the same relevance to perceptual (visual, auditory, olfactory and taste perceptions) and functional (tactual, motor and language-mediated) information, and in the representation of artefacts both males and females gave a greater weight to functional than to perceptual features. Although the greater relevance of functional information in the representation of artefacts was expected, the equal weight of perceptual and functional information in the representation of biological entities was not. This finding, which is consistent with some results (e.g. [13,18,33]) but not with others (e.g. [14,30]), can probably be explained by methodological reasons (see [17]), but in any case is not relevant to the issue discussed here. What is relevant is that, contrary to the Bermeitinger et al.'s [4] hypothesis, the weight of 'perceptual' and 'functional' features was very similar in males and females in the representation of biological categories. Several explanations can be advanced to account for the apparent contrast between our results, and those obtained by Bermeitinger et al. [4]. The first explanation refers to the different acceptation in which the term functional information was

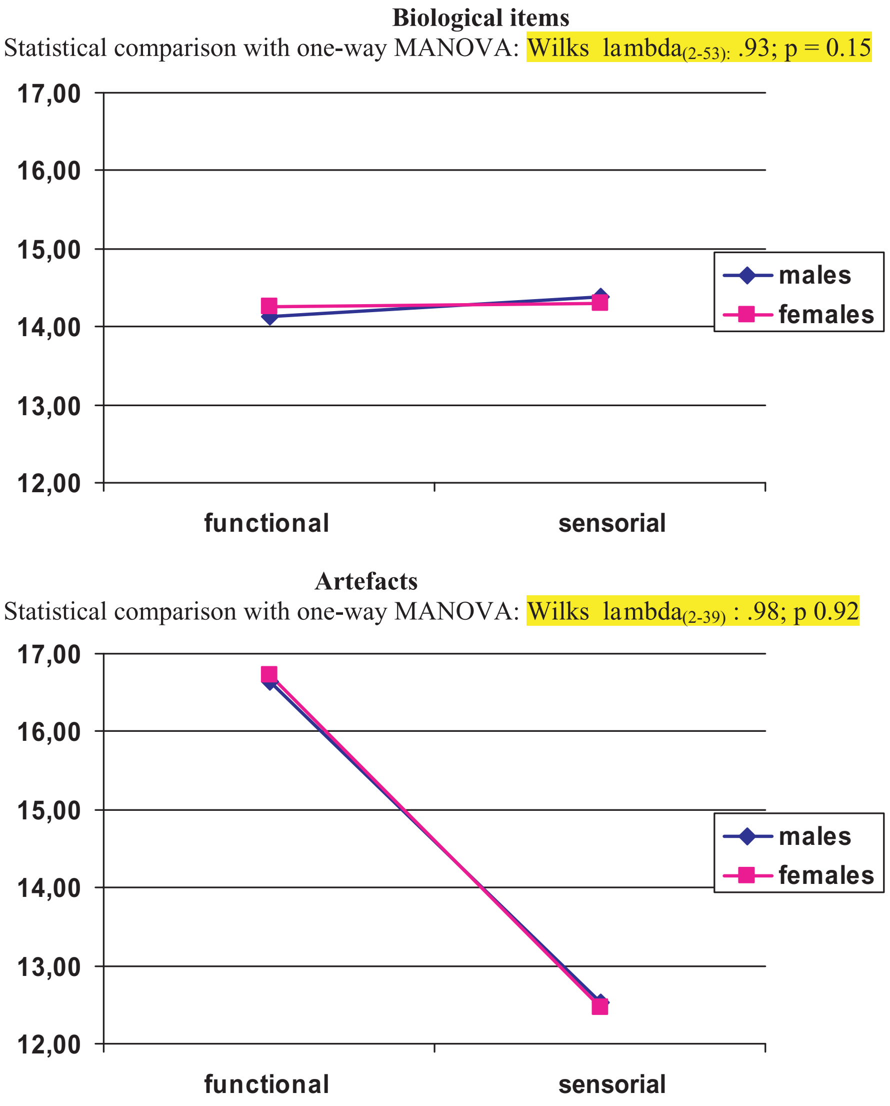

Fig. 1. Weight attributed by men and women to ‘perceptual’ (visual, auditory, gustatory and olfactory) and ‘functional’ (somato-sensory, motor and language-mediated) sources of knowledge in the representation of biological and artefact stimuli included in our study.

used in the two inquiries because in the Bermeitinger et al.’s study this term specifically concerned the function of an object (e.g. if a candle and an electric bulb have the same function) whereas in our investigation it included both functional information and other types of ’language-mediated encyclopaedic information’. This explanation is, in our opinion, unlikely for two reasons: the first is that the Bermeitinger et al. study focused attention on the perceptual (rather than on the ‘functional’ features or on the functional/perceptual ratio) and in our study we found no difference between males and females when perceptual features were taken into account; the second is that no trend in the expected direction was found in any of the analyses undertaken.

The second explanation could refer to the different requests of investigations evaluating respectively the weight that different sources of knowledge could have in the mental representation of biological and artefact categories and the structure of semantic memory, investigated using a semantic priming paradigm. There might be two (a conflicting and a more conciliatory) versions of this interpretation. The first, more conflicting, version assumes that the Bermeitinger et al.’s [4] hypothesis was correct, but we were unable to answer questions regarding the topic investigated in that study because of the procedure we chose. According to this viewpoint, the explicit requests used in our study to assess the sources of knowledge subsuming different semantic categories in men and women did not allow to explore (as an implicit task would have done) the structure of semantic memory and the different processes within semantic memory that distinguish men from women. The participants in our study therefore went through their memory and checked the relevance of each dimension, without necessarily exploring the different structures of semantic memory and processes within semantic memory, that should be focused on ‘perceptual’ features in women and both on ‘functional’ and on ‘perceptual’ features in men. According to this interpretation, the Bermeitinger et al.’s [4] hypothesis was, therefore, correct, but the methods that we used to check it were not appropriate.

The second (more conciliatory) hypothesis assumes that the data by Bermeitinger et al. [4] and those of our explicit task can be perfectly integrated, because in the implicit task used by these authors, default settings of the processing which differed between males and females could be changed by focusing on perceptual vs. functional features (see Experiment 2 of Bermeitinger et al. [4]). This is especially interesting because it suggests that the underlying fixed memory structures do not differ between females and males and that only the mode of processing a target with the help of a prime (as a result of experience and/or of learning processes) differed between men and women. This second interpretation is consistent with the data of the literature, reported in the introduction, which showed a sex-related dissociation between ‘animals’ and ‘plant life’, because it acknowledges the importance of social-role related familiarity factors in modulating the ability of men and women to process members of various semantic categories. A further support to this interpretation also comes by the fact that, just as men are superior for one category of living beings (animals), women perform better with some kinds of artefacts, such as ‘furniture’ (e.g. [1,27]) and ‘kitchen utensils’ (e.g. [31]) with which they are more familiar. Obviously, the advantage of men for animals and of women for ‘furniture’ and ‘kitchen utensils’ are at variance with the Bermeitinger et al.’s hypothesis, assuming that gender-related asymmetries may be due to a tendency of females to focus mainly on perceptual features (considered as more relevant for the representation of living beings) and of males to focus equally on perceptual and functional features.

A last finding that we would shortly consider, at the end of this discussion, concerns the fact that in our study we found no difference between males and females in their familiarity with any of the subcategories taken into account, just as in the superordinate ‘biological’ or ‘artefact’ categories. This unexpected finding can, in our opinion, be explained by considering the nature of the male and female samples included in our study, namely the fact that they were undergraduate students belonging to a generation in which the traditional social roles have almost completely disappeared. An argument in favour of this point comes from results recently obtained by Moreno-Martinez et al. [30], studying semantic fluency in a large series of categories (7 living and 7 nonliving) in male and female AD patients, and in elderly and young controls. Rather than on results obtained on AD patients (that cannot be easily interpreted, due to the coexistence of normal and pathological factors) we would focus attention on the fact that in the young group (as in our study) these authors found no difference between males and females on either the living or the non-living subcategories, whereas in the elderly group they observed differences consistent with the importance of social-role related familiarity factors. Elderly females showed, indeed, greater fluency for flowers, vegetables and kitchen utensils, whereas elderly males showed better fluency for musical instruments. We think, therefore, that, even if further investigations are certainly required to clarify this complex issue, the importance of social-role related familiarity factors to explain the differences between males and females in the ability to process different semantic categories cannot be easily dismissed.

## Appendix 1

List of the fruits (items 1–5), vegetables (6–10), flowers (11–16), domestic animals (17–22), wild animals (23–28), tools (29–34), pieces of furniture (35–39), articles of clothing (40–44) and vehicles (45–49) used in our study

| | |
|---|---|
| 1. banana | banana |
| 2. arancia | orange |
| 3. pera | pear |
| 4. pesca | peach |
| 5. uva | grapes |
| 6. zucchine | courgette |
| 7. peperoni | pepper |
| 8. pomodori | tomato |
| 9. carote | carot |
| 10. carciofo | artichoke |
| 11. geranio | geranium |
| 12. tulipano | tulip |
| 13. rosa | rose |
| 14. giglio | lily |
| 15. margherita | marguerite |
| 16. garofano | carnation |
| 17. coniglio | rabbit |
| 18. cavallo | horse |
| 19. gallo | cock |
| 20. maiale | pig |
| 21. gatto | cat |
| 22. cane | dog |
| 23. leone | leon |
| 24. cammello | camell |
| 25. scimmia | monkey |
| 26. giraffa | giraffe |
| 27. tigre | tiger |
| 28. elefante | elephant |
| 29. forchetta | fork |
| 30. ombrello | umbrella |
| 31. forbici | scissor |
| 32. martello | hammer |
| 33. cucchiaino | teaspoon |
| 34. pettine | comb |
| 35. armadio | wardrobe |
| 36. letto | bed |
| 37. tavolo | table |
| 38. sedia | chair |
| 39. specchio | mirror |
| 40. calzino | sock |
| 41. maglione | sweater |
| 42. cappello | hat |
| 43. giacca | coat |
| 44. scarpa | shoe |
| 45. barca | boat |
| 46. nave | ship |
| 47. macchina | car |
| 48. treno | train |
| 49. aeroplano | airplane |

## Appendix 2

Instructions given to subjects participating in our study

Your task will consist of indicating, on a scale ranging between 0 and 7, your own familiarity with a series of objects and the relevance that in their mental representations could play a number of modalities of information that we can call ‘sources of knowledge’.

When giving your evaluation, make a cross in the appropriate box, where 0 corresponds to ‘no familiarity’ or ‘no relevance’, whereas 7 corresponds to ‘very high familiarity’ or ‘very high relevance’

*Example:*

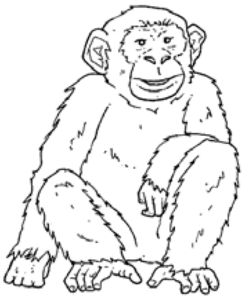

**MONKEY**

| **Familiarity** | 0 | 1 | 2 | 3 | 4 | 5 | 6 | 7 |
|---|---|---|---|---|---|---|---|---|

← Lower familiarity Higher familiarity →

| **Sources of Knowledge** | | | | | | | | |
|---|---|---|---|---|---|---|---|---|
| **Visual** | 0 | 1 | 2 | 3 | 4 | 5 | 6 | 7 |
| **Auditory** | 0 | 1 | 2 | 3 | 4 | 5 | 6 | 7 |
| **Olfactory** | 0 | 1 | 2 | 3 | 4 | 5 | 6 | 7 |
| **Gustatory** | 0 | 1 | 2 | 3 | 4 | 5 | 6 | 7 |
| **Tactual** | 0 | 1 | 2 | 3 | 4 | 5 | 6 | 7 |
| **Action mediated** | 0 | 1 | 2 | 3 | 4 | 5 | 6 | 7 |
| **Language mediated** | 0 | 1 | 2 | 3 | 4 | 5 | 6 | 7 |

←Lower relevance Higher relevance→
